# Association of Modifiable Risk Factors With Dental Caries Among Indigenous and Nonindigenous Children in Australia

**DOI:** 10.1001/jamanetworkopen.2019.3466

**Published:** 2019-05-03

**Authors:** Xiangqun Ju, Loc Do, Diep Ha, Lisa Jamieson

**Affiliations:** 1 Australian Research Centre for Population Oral Health, University of Adelaide, Adelaide, Australia

## Abstract

**Importance:**

Although the prevalence of untreated dental caries among Indigenous Australian children greatly exceeds the prevalence observed among non-Indigenous children, the associations of dental caries with risk factors is considered to be the same.

**Objective:**

To estimate the association of modifiable risk factors with area-based inequalities in untreated dental caries among Indigenous and non-Indigenous Australian children using decomposition analysis.

**Design, Setting, Participants:**

Cross-sectional study using data from Australia’s National Child Oral Health Study 2012-2014, a nationally representative sample of both Indigenous and non-Indigenous children aged 5 to 14 years. Data analyses were completed in November 2018.

**Main Outcomes and Measures:**

Outcomes were the mean number of decayed tooth surfaces in the primary dentition for children aged 5 to 10 years and mean number of decayed tooth surfaces in the permanent dentition for children aged 8 to 14 years. The area-based measure was the school-based Index of Community Socio-Educational Advantage, with individual-level variables including sex, equivalized household income, tooth-brushing frequency, sugar-sweetened beverage (SSB) consumption, time from last dental visit, and residing in an area with water fluoridation.

**Results:**

There were 720 Indigenous children aged 5 to 10 years, 14 769 non-Indigenous children aged 5 to 10 years, 738 Indigenous children aged 8 to 14 years, and 15 631 non-Indigenous children aged 8 to 14 years. For area-based inequalities in primary dentition among Indigenous children, two-thirds of the contribution was associated with SSB consumption (65.9%; 95% CI, 65.5%-66.3%), followed by irregular tooth brushing (15.0%; 95% CI, 14.6%-15.5%) and low household income (14.5%; 95% CI, 14.1%-14.8%). Among non-Indigenous children, almost half the contribution was associated with low household income (47.6%; 95% CI, 47.6%-47.7%), followed by SSB consumption (31.0%; 95% CI, 30.9%-31.0%) and residing in an area with nonfluoridated water (9.5%; 95% CI, 9.5%-9.6%). For area-based inequalities in permanent dentition among Indigenous children, 40.0% (95% CI, 39.9%-40.1%) of the contribution was associated with residing in an area with nonfluoridated water, followed by low household income (20.0%; 95% CI, 19.7%-20.0%) and consumption of SSBs (20.0%; 95% CI, 19.9%-20.1%). Among non-Indigenous children, the contribution associated with low household income, SSB consumption, and last dental visit more than a year ago were each 28.6%.

**Conclusions and Relevance:**

The association of modifiable risk factors with area-based inequalities in untreated dental caries among Indigenous and non-Indigenous Australian children differed substantially. Targets to reduce SSB consumption may reduce oral health inequalities for both groups; however, Indigenous children require additional focus on oral hygiene.

## Key Points

**Question:**

Does the contribution of modifiable risk factors on area-based inequalities in untreated dental caries among Australian children differ by Indigenous status?

**Findings:**

In this cross-sectional study of Australian children, the association of modifiable risk factors with area-based inequalities in untreated dental caries among Indigenous and non-Indigenous Australian children differed substantially. Consumption of sugar-sweetened beverages was associated with dental caries for both groups, and irregular tooth brushing was also significantly associated with dental caries for Indigenous children.

**Meaning:**

Targets to reduce consumption of sugar-sweetened beverages may reduce oral health inequalities for both groups; however, Indigenous children require additional focus on oral hygiene.

## Teaser

This cross-sectional study estimates the association of modifiable risk factors with dental caries in Indigenous and non-Indigenous Australian children using data from the National Child Oral Health Study 2012-2014.

## Introduction

Indigenous children in Australia (those identifying as Aboriginal or Torres Strait Islander or both) experience profoundly greater inequalities on almost every indicator of health and well-being compared with their non-Indigenous peers.^1^ There is a higher prevalence of nutrition-associated stunting, nonoptimal blood pressure growth outcomes,^2^ and poorer social and emotional well-being.^3^ Approximately one-fifth of Aboriginal children are overweight or obese^4^ and approximately 30% may not be exercising at recommended levels.^3^ Little is known about the dietary patterns of Aboriginal children, but there is some evidence of low rates of fruit, vegetable, water, and milk consumption.^5^ An Aboriginal child who has been forcibly removed from their family as a child or has a primary carer who has had contact with the mental health system, is not the child’s biological relative, or is single has a higher risk of poor health and developmental outcomes.^6^ The literature suggests that many of the conditions experienced in Aboriginal childhood are antecedents to chronic disease in later life.^6^

Regrettably, these inequalities also extend to oral health. In Australia’s National Child Oral Health Study 2012-2014, 44% of Indigenous children had 1 or more deciduous teeth with untreated dental caries, compared with 26% of non-Indigenous children.^7^ Other research has indicated that Indigenous children in some areas have up to 5 times the prevalence of dental disease of their non-Aboriginal counterparts.^8^ Lack of access to culturally responsive dental health professionals is frequently cited as a reason for this inequity, together with specific behavioral risk factors and social determinants.^9^

It is widely accepted that dental disease is socially patterned, with a plethora of research indicating that socially disadvantaged populations have substantially greater experience of dental disease than those who are more advantaged for both individual and area-based measures of inequality.^10,11^ Among children, the literature suggests a number of risk factors for experience of dental caries, which conceptually can be grouped as related to sociodemographic characteristics, dental hygiene, diet, dental service use, and environment (water fluoridation). Sociodemographic factors include household income, which is a proxy for broader material influences that play in a role in oral health literacy, purchasing power, and general health education.^12^ Oral hygiene practices such as tooth-brushing frequency are associated with the physical removal of food and beverage residue in the oral cavity, lowering levels of *Streptococcus mutans* through reduction of plaque,^13^ and bolstering fluoride in saliva reservoirs if fluoridated toothpaste is used. Diet, specifically a cariogenic diet, is associated with dental caries, with recent evidence demonstrating that frequent consumption of sugar-sweetened beverages (SSBs) is one of the strongest associations.^14^ Frequent use of dental services provides preventive measures such as application of topical fluoride and general prophylaxis and enables any untreated dental caries to be restoratively managed.^15^ Consumption of fluoridated water is one of the most successful public health interventions regarding oral health and is especially beneficial in reducing oral health inequalities between Indigenous and non-Indigenous Australian children.^16^

While these factors associated with dental disease have been identified, to our knowledge, there has been no application of analysis that enables specific percentages of risk contribution to be calculated. Nor has there been any analysis of whether the risks, and contribution of these risks, might differ between Indigenous and non-Indigenous children. This is important for both targeting of effective oral health promotion initiatives and for policy implications in the allocation of scarce resources in the dental public health setting. This study, therefore, aims to analyze both area- and individual-level oral health inequalities among Australian children and, specifically, to assess the association of individual-level sociodemographic, oral hygiene, dietary, dental service use, and water fluoridation factors with area-based inequalities in untreated dental caries, stratified by Indigenous status. The hypothesis is that the percentage association of each factor will differ for Indigenous and non-Indigenous children.

## Methods

This study is reported according to the Strengthening the Reporting of Observational Studies in Epidemiology (STROBE) reporting guideline. The study was reviewed and approved by the University of Adelaide Human Research Ethics Committee; research ethics committees within each jurisdiction; the 3 education sectors: public, independent, and Catholic schools; and the Indigenous Human Research Ethics Committee at the Menzies Institute. Parents provided signed, informed consent for their child to participate.

### Sampling

Data were obtained from Australia’s National Child Oral Health Study 2012-2014 (NCOHS), a population-based cross-sectional survey of Australian children aged 5 to 14 years from both primary and secondary schools, for which data collection details have been previously described.^7^ Briefly, NCOHS used a 2-stage stratified sample design to draw a representative sample of children across Australia. In the first stage, a sampling school was created from a list provided by each jurisdiction, which included all public, Catholic, and independent primary and secondary schools. Schools were then selected with a probability proportional to size of enrollment. In the second stage, a cluster of children was randomly sampled from each participating school. Parents of selected children were approached to participate. Participation included completion of the parental questionnaire and an oral epidemiological examination of the children by trained and calibrated examiners. Dental examinations included assessments of primary and permanent caries at the tooth surface level. The criteria and methods for the assessment of caries were based on the US Third National Health and Nutrition Examination Survey and the Australian Research Centre for Population Oral Health’s Child Dental Health Surveys. All examiners were tested in the field against 1 of 2 senior trainers to estimate interexaminer reliability. The intraclass correlation coefficient (ICC) for caries assessment scores ranged from 0.67 to 0.99, indicating good to excellent reliability.

### Weighting

Child examination and questionnaire data were weighted separately for each state and territory by deriving survey weights, which were adjusted by school type and sociodemographic characteristics of participating children. Population estimates derived from the weighted sample closely reflected the true child population.^7^

### Variables

#### Outcome Variable

The outcome variable was mean number of tooth surfaces with untreated decay for the primary dentition (ds) (age 5-10 years) and for the permanent dentition (DS) (age 8-14 years).

#### Area-Based Explanatory Variable

The area-based measure was the school-based Index of Community Socio-Educational Advantage (ICSEA), a composite variable of school socioeconomic status that combines student characteristics (parent occupation and level of education) and school-area characteristics (proportion of Indigenous students and geographical location).^17^ School level was dichotomized into low (ICSEA score <986; most disadvantaged) and high (ICSEA score ≥986; most advantaged).

#### Individual-Level Explanatory Variables

Individual-level explanatory variables included sociodemographic, dental hygiene, diet, dental service use, and environmental factors. Sociodemographic factors included child sex and equivalized household income. Equivalized household income was derived by calculating an equivalence factor according to the modified Organisation for Economic Co-operation and Development equivalence scale, then dividing income by the factor. The equivalence factor was built up by allocating points to each person in a household (1 point to the first adult, 0.5 points to each additional person aged ≥15 years, and 0.3 points to each child aged <15 years) and then summing the equivalence points of all household members.^18^ Equivalized household income was grouped into 4 approximately equal quartiles, with quartile 1 being lowest and quartile 4 being highest. The dental hygiene variable included frequency of tooth brushing (<2 times per day and ≥2 times per day). The diet variable was number of glasses of SSB per day (≥2 SSB, 1 SSB, or 0 SSB). Dental service use included time since last dental visit (≥18 months or <18 months). Environmental factors included exposure to water fluoridation (yes for ≥0.5 mg/L fluoride vs no for <0.5 mg/L fluoride). Fluoridation status of the local water supply was based on the child’s residential postcode. The Australian Research Centre for Population Oral Health maintains a postcode-level database of fluoride concentration in water supplies. The recommended range for fluoride in water supplies in Australia is 0.6 to 1.1 mg/L dependent on ambient temperature. If the fluoride level in the drinking water was at least 0.5 mg/L, the water was defined as fluoridated; otherwise, it was classified as nonfluoridated.

### Statistical Analysis

Data were analyzed separately for Indigenous and non-Indigenous children for age groups 5 to 10 years and 8 to 14 years, which included descriptive multilevel multivariable regression, Blinder-Oaxaca, and Neumark decomposition analyses. Data analyses were completed in November 2018. The mean ds and DS were estimated according to each explanatory variable using a procedure in SUDAAN statistical software version 11.0.3 (RTI International). Multilevel general linear regressions were used to build 2-level models in which individuals (children, level 1) were nested within schools (ICSEA, level 2). The random component (level 1 and 2 variances) and the fixed component (regression coefficients) of the models were estimated by using maximum likelihood to test the association between school level and untreated decayed surfaces after adjusting for individual-level explanatory variables. The ICC was estimated as the percentage of school-level variance in the total (both individual and school) variance to determine whether mean ds and DS vary notably across schools (ICC > 0):

ICC = τ_00_/(τ_00_+ ϭ2)

where ϭ2 is the average variance of individual’s (child’s) ds or DS within schools and τ_00_ quantifies the variation in mean ds and DS across schools. The ICC value indicates the proportion of variance at the school level.^19^

The models were compared using an Akaike information criterion to assess goodness of fit and to determine which models were preferred. The lower values of Akaike information criterion indicate a better model fit. All models were developed and fitted using SAS statistical software version 9.4 (SAS Institute Inc).

Blinder-Oaxaca and Neumark decomposition analyses, straightforward statistical methods that use, in our case, socioeconomic area as a way to explain observed differences between groups (*Z* test with 2-tailed *P* < .05), were used to identify factors that explained most of the school-level inequalities in untreated dental caries using STATA version 14 statistical software (StataCorp LLC). Analyses used the surveys’ sampling probability weights, accounting for the sampling strategy and nonresponse, to provide population-representative data.

## Results

A total of 720 Indigenous and 14 769 non-Indigenous children aged 5 to 10 years and 738 Indigenous and 15 631 non-Indigenous children aged 8 to 14 years were included. Table 1 shows the sample characteristics and mean number of untreated decayed tooth surfaces. Among children aged 5 to 10 years, 74.6% of Indigenous children attended schools in the lowest ICSEA level compared with 31% of non-Indigenous children. More than half of Indigenous children (56.3%) resided in homes with the lowest household income, compared with approximately 28% of non-Indigenous children. Nearly half of Indigenous children (47.6%) brushed less than twice per day compared with 31.1% of non-Indigenous children. A total of 42.0% of Indigenous children consumed 2 or more cups of SSB per day, compared with 19.6% of non-Indigenous children. Approximately one-third (35.0%) of Indigenous children had a last dental visit more than 18 months previous, compared with 27.3% of non-Indigenous children. A total of 42.2% of Indigenous children resided in areas without water fluoridation, compared with 28.8% of non-Indigenous children. The mean ds for Indigenous children was nearly 3 times that of non-Indigenous children (3.5 vs 1.2). The highest levels of mean ds among Indigenous children were observed among those in the lowest school ICSEA level (4.3), those in the lowest household income quartile (3.8), those brushing less than twice daily (4.4), those consuming more than 2 SSBs per day (5.0), those who last received dental care more than 18 months previously (5.0), and those who did not reside in areas with fluoridated water (4.1).

**Table 1.** Sample Characteristics and Mean Number of Untreated Decay Between Indigenous and Non-Indigenous Children Aged 5 to 14 Years (Weighted)

Characteristic	Weighted % (95% CI)		Mean ds or DS, No. (95% CI)		Ratio of Indigenous to Non-Indigenous
	Indigenous	Non-Indigenous	Indigenous	Non-Indigenous	
**Aged 5-10 y (n = 15 489)**					
Total	100	100	3.5 (2.5-4.5)	1.2 (1.0-1.3)	2.9
Index of Community Socio-Educational Advantage school level					
Lower: <986	74.6 (66.1-81.5)	30.6 (26.2-35.5)	4.3 (3.1-5.6)	1.7 (1.4-2.0)	2.5
Higher: ≥986	25.4 (18.5-33.9)	69.4 (64.5-73.8)	1.2 (0.6-1.9)	0.9 (0.8-1.4)	1.3
Sex					
Boy	50.5 (44.9-56.2)	51.6 (50.0-53.1)	3.9 (2.4-5.4)	1.2 (1.1-1.4)	3.3
Girl	49.5 (43.8-55.1)	48.4 (46.9-50.0)	3.1 (2.1-4.2)	1.1 (0.9-1.2)	2.8
Equivalized household income^a^					
Quartile 1: lowest	56.3 (50.9-65.4)	27.9 (25.6-30.3)	3.8 (2.5-5.1)	1.9 (1.6-2.2	2.0
Quartile 2	19.2 (14.9-24.4)	22.4 (21.1-23.8)	1.5 (0.5-2.4)	1.1 (0.9-1.3)	1.4
Quartile 3	14.8 (10.3-20.8)	21.8 (20.5-23.2)	1.1 (0.5-1.7)	0.7 (0.6-0.8)	1.6
Quartile 4: highest	7.6 (4.6-12.4)	27.9 (25.4-30.6)	0.2 (0-0.4)	0.6 (0.5-0.7)	0.3
Tooth-brushing frequency					
<2 Times per day	47.6 (41.0-54.2)	31.1 (29.6-32.7)	4.4 (3.2-5.6)	1.6 (1.3-1.9)	2.8
≥2 Times per day	52.4 (45.8-59.0)	68.9 (67.3-70.4)	2.0 (1.1-2.8)	0.9 (0.8-1.0)	2.2
Daily consumption sugar sweetened beverage					
≥2 Cups	42.0 (34.4-49.9)	19.6 (18.0-21.3)	5.0 (3.2-6.9)	2.4 (1.9-2.8)	2.1
1-1.9 Cups	32.9 (27.1-39.4)	29.6 (28.2-30.9)	2.5 (1.2-3.8)	1.1 (1.0-1.3)	2.3
0 Cups	25.1 (19.7-31.4)	50.8 (48.6-53.0)	2.3 (1.1-3.5)	0.7 (0.6-0.7)	3.3
Last dental visit					
≥18 mo	35.0 (30.0-40.3)	27.3 (25.3-29.4)	5.0 (3.2-6.7)	1.7 (1.4-2.0)	2.9
<18 mo	65.0 (59.7-70.0)	72.7 (70.6-74.7)	2.8 (1.9-3.6)	1.0 (0.9-1.1)	2.8
Water fluoridated area					
Yes: ≥0.5 mg/L	57.8 (48.3-66.8)	71.2 (68.0-74.2)	3.3 (2.1-4.5)	1.1 (0.9-1.2)	3.0
No: <0.5 mg/L	42.2 (33.2-51.7)	28.8 (25.8-32.0)	4.1 (2.3-6.0)	1.4 (1.1-1.6)	2.9
**Aged 8-14 y (n = 16 669)**					
Total	100	100	0.8 (0.6-1.1)	0.3 (0.2-0.3)	2.7
Index of Community Socio-Educational Advantage school level					
Lower: <986	70.6 (62.8-77.3)	32.0 (28.5-35.9)	1.0 (0.6-1.1)	0.4 (0.4-0.5)	2.5
Higher: ≥986	29.4 (22.8-37.3)	68.0 (64.1-71.6)	0.4 (0.1-0.7)	0.2 (0.1-0.2)	2.0
Sex					
Boy	49.0 (44.1-53.9)	51.2 (49.4-53.0)	0.7 (0.4-0.9)	0.2 (0.2-0.3)	3.5
Girl	51.0 (46.1-55.9)	48.8 (47.0-50.6)	0.9 (0.6-1.3)	0.3 (0.2-0.3)	3.0
Equivalized household income^a^					
Quartile 1: lowest	58.9 (52.2-65.3)	28.7 (23.8-26.3)	0.8 (0.5-1.1)	0.4 (0.3-0.5)	2.0
Quartile 2	21.5 (16.8-27.0)	25.0 (23.8-26.3)	0.6 (0.2-1.0)	0.2 (0.2-0.3)	3.0
Quartile 3	14.4 (10.3-19.8)	21.2 (20.1-22.3)	0.3 (0-0.6)	0.2 (0.1-0.2)	1.5
Quartile 4: highest	5.2 (3.2-8.4)	25.1 (23.3-27.0)	0 (0-0)	0.1 (0.1-0.1)	0.0
Tooth-brushing frequency					
<2 Times per day	49.0 (43.2-54.9)	30.0 (28.6-31.4)	0.8 (0.5-1.2)	0.4 (0.3-0.4)	2.0
≥2 Times per day	51.0 (45.1-56.8)	70.0 (68.6-71.4)	0.7 (0.5-0.9)	0.2 (0.2-0.2)	3.5
Daily consumption sugar sweetened beverage					
≥2 Cups	49.4 (43.4-55.3)	26.0 (24.5-27.6)	1.2 (0.8-1.5)	0.5 (0.4-0.6	2.4
1-1.9 Cups	28.4 (23.9-33.4)	32.4 (31.1-33.6)	0.7 (0.3-1.1)	0.2 (0.2-0.3)	3.5
0 Cups	22.2 (17.5-27.8)	41.6 (39.9-43.4)	0.2 (0.1-0.3)	0.1 (0.1-0.2)	2.0
Last dental visit					
≥18 mo	27.1 (22.8-31.9)	17.9 (16.5-19.4)	0.9 (0.5-1.4)	0.5 (0.4-0.7)	1.8
<18 mo	72.9 (68.1-77.2)	82.1 (80.6-83.5)	0.8 (0.5-1.1)	0.2 (0.2-0.2)	4.0
Water fluoridated area					
Yes: ≥0.5 mg/L	56.7 (47.8-65.3)	70.7 (68.3-73.1)	0.6 (0.3-0.9)	0.2 (0.2-0.3)	3.0
No: <0.5 mg/L	43.3 (34.7-52.2)	29.3 (26.9-31.7)	1.1 (0.7-1.5)	0.3 (0.3-0.4)	3.7

Abbreviations: ds, decay of primary dentition surface; DS, decay of permanent dentition surface.

^a^ Missing values for equivalized household income: all children, 13.5%; Indigenous children, 29.7%; non-Indigenous children, 11.7%.

Among children aged 8 to 14 years, 70.6% of Indigenous children attended schools in the lowest ICSEA level compared with 32.0% of non-Indigenous children. More than half of Indigenous children (58.9%) resided in homes with the lowest household income, compared with 28.7% of non-Indigenous children. Nearly half of Indigenous children (49.0%) brushed less than twice per day compared with 30.0% of non-Indigenous children. Approximately half of Indigenous children (49.4%) consumed 2 or more cups of SSB per day, compared with 26.0% of non-Indigenous children. Among Indigenous children, 27.1% had last visited a dentist more than 18 months previous, compared with 17.9% of non-Indigenous children. In all, 43.3% of Indigenous children resided in areas without water fluoridation, compared with 29.3% of non-Indigenous children. The mean DS for Indigenous children was nearly 3 times that of non-Indigenous children (0.8 vs 0.3). The highest levels of mean DS among Indigenous children were observed among those in the lowest school ICSEA level (1.0), those in the lowest household income quartile (0.8), those brushing less than twice daily (0.8), those consuming more than 2 SSBs per day (1.2), those who last received dental care more than 18 months previously (0.9), and those who did not reside in areas with fluoridated water (1.1).

Table 2 presents the multilevel analyses on untreated decay surfaces among Indigenous and non-Indigenous children by the area-based ICSEA measure. In the null models, Indigenous children attending disadvantaged schools had 2.42 (model 2) and 0.48 (model 6) times higher mean ds and mean DS, respectively, than Indigenous children attending more advantaged schools. Non-Indigenous children attending disadvantaged schools had 0.57 (model 10) and 0.17 (model 14) times higher mean ds and mean DS, respectively, than non-Indigenous children attending more advantaged schools. For Indigenous children, these differences were attenuated in the final models (model 4 and model 8), after adjustment for individual-level explanatory variables. The ICC values decreased from null to final models for both Indigenous children (11.8% to 11.3% for mean ds and 10.3% to 7.7% for mean DS) and non-Indigenous children (13.5% to 8.7% for mean ds and 13.8% to 8.3% for mean DS). The Akaike information criterion values decreased from the null to the full models for both Indigenous and non-Indigenous children across both age groups, indicating that multilevel analysis was the best analytical approach for this data.

**Table 2.** Multilevel Analyses on Untreated Decayed Surfaces Among Australian Indigenous and Non-Indigenous Children

Characteristic	Model 1^a^	Model 2^a^	Model 3^a^	Model 4^a^
**Indigenous, Aged 5-10 y**				
Estimates (SE)				
Intercept, τ_00_	6.6 (1.4)	5.9 (1.3)	5.7 (1.3)	3.5 (1.3)
Residual, ϭ2	49.3 (2.8)	48.87 (2.8)	48.1 (2.8)	27.7 (2.3)
ICSEA school level estimates (95% CI)				
Low	NA	2.4 (1.2 to 3.7)	2.4 (1.2 to 3.7)	1.17 (−0.03 to 2.37)
High	NA	0	0	0
Level 2 variance: ICC, %^b^	11.8	10.7	10.7	11.3
Model fit: AIC	4892	4877	4877	2515
**Non-Indigenous, Aged 5-10 y**				
Estimates (SE)				
Intercept	1.2 (0.1)	1.1 (0.1)	1.1 (0.1)	0.65 (0.1)
Residual	7.8 (0.1)	7.8 (0.1)	7.9 (0.1)	6.8 (0.1)
ICSEA school level estimates (95% CI)				
Low	NA	0.6 (0.4 to 0.8)	0.5 (0.35 to 0.7)	0.3 (0.1 to 0.5)
High	NA	0	0	0
Level 2 variance: ICC, %^b^	13.5	12.3	12.0	8.7
Model fit: AIC	76 508	76 478	74 606	55 606
**Indigenous, Aged 8-14 y**				
Estimates (SE)				
Intercept, τ_00_	0.6 (0.1)	0.5 (0.1)	0.5 (0.1)	0.2 (0.1)
Residual, ϭ2	4.9 (0.3)	4.9 (0.3)	5.0 (0.3)	2.15 (0.2)
ICSEA school level estimates (95% CI)				
Low	NA	0.5 (0.1 to 0.8)	0.45 (0.1 to 0.8)	0.2 (−0.1 to 0.5)
High	NA	0	0	0
Level 2 variance: ICC, %^b^	10.3	9.6	9.8	7.7
Model fit: AIC	3279	3274	3161	1686
**Non-Indigenous, Aged 8-14 y**				
Estimates (SE)				
Intercept	0.11 (0.01)	0.10 (0.01)	0.10 (0.01)	0.05 (0.01)
Residual	0.69 (0.01)	0.69 (0.01)	0.69 (0.01)	0.55 (0.01)
ICSEA school level estimates (95% CI)				
Low	NA	0.2 (0.1 to 0.2)	0.7 (0.1 to 0.2)	0.1 (0.1 to 0.15)
High	NA	0	0	0
Level 2 variance: ICC, %^b^	13.8	12.7	12.7	8.3
Model fit: AIC	42 250	42 208	41 140	28 550

Abbreviations: AIC indicates Akaike information criterion; ICC, intraclass correlation coefficient; ICSEA, Index of Community Socio-Educational Advantage; NA, not applicable.

^a^ Model 1 was the intercept-only (null) model adjusted for child’s sex and mean centered age (= mean-age). Model 2 included the null models plus school-level disadvantage (ICSEA). Model 3 included previous models plus water fluoridated area. Model 4 included previous models plus equivalized household income, sugar sweetened beverage consumption, tooth-brushing frequency, and last dental visit.

^b^ ICC = τ_00_/(τ_00_+ ϭ2).

The decomposition models (Table 3 and Table 4) demonstrated that, for area-based inequalities in untreated ds among Indigenous children, two-thirds of the contribution was associated with SSB consumption (65.9%; 95% CI, 65.6%-66.2%), followed by irregular tooth brushing (15.0%; 95% CI, 14.6%-15.5%) and low household income (14.5%; 95% CI, 14.1%-14.8%). Among non-Indigenous children, almost half the contribution was associated with low household income (47.6%; 95% CI, 47.5%-47.6%), followed by SSB consumption (31.0%; 95% CI, 30.9%-31.0%) and residing in an area with nonfluoridated water (9.5%; 95% CI, 9.5%-9.6%). For area-based inequalities in untreated DS among Indigenous children, 40.0% (95% CI, 39.9%-40.1%) of the contribution was associated with residing in an area with no water fluoridation followed by low household income (20.0%; 95% CI, 19.7%-20.0%) and consumption of SSBs (20.0%; 95% CI, 19.9%-20.1%). Among non-Indigenous children, the contribution associated with low household income, SSB consumption, and last dental visit more than a year ago was 28.9% for each.

**Table 3.** Decomposition of Contributors to School-Level (Low vs High) Effect on Observed Gap in Untreated Decay Among Australian Children

Variable	Age 5-10 y		Age 8-14 y	
	Indigenous	Non-Indigenous	Indigenous	Non-Indigenous
Mean ds or DS (low)	3.60	1.64	0.72	0.41
Mean ds or DS (high)	1.43	0.85	0.25	0.15
Raw difference between 2 school levels	2.17	0.79	0.47	0.26
Due to endowments	0.08	0.41	0.10	0.08
Due to coefficients	1.11	0.12	0.26	0.05
Due to interaction	0.98	0.26	0.12	0.13

Abbreviations: ds, decay of primary dentition surface; DS, decay of permanent dentition surface.

**Table 4.** Explained Component of Contributors Between School-level (Low vs High) on Observed Gap in Untreated Decay Among Indigenous Children (Decomposition Analysis)

Variable	Age 5-10 y								Age 8-14 y							
	Indigenous				Non-Indigenous				Indigenous				Non-Indigenous			
	E	C	I^a^	Proportion Explained, % (95% CI)	E	C	I^a^	Proportion explained, % (95% CI)	E	C	I^a^	Proportion Explained, % (95% CI)	E	C	I^a^	Proportion Explained, % (95% CI)
Sex	−0.01	−3.86	−0.01	0.6 (0.4 to 0.7)	0	−0.70	0	0 (0 to 0)	0.01	0.12	−0.01	10.0 (9.96 to 10.04)	−0.000.68	0.01	0	0 (0 to 0)
Equivalized household income^b^	0.23	1.38	−0.25	14.5 (14.1 to 14.8)	0.20	0.16	−0.21^c^	47.6 (47.5 to 47.7)	0.02	−0.17	−0.02	20.0 (19.7 to 20.0)	0.02	0.15	−0.03^c^	28.6 (28.5 to 29.6)
Tooth-brushing frequency	−0.05	1.71	−0.26	15.0 (14.6 to 15.5)	0.03	0.10	−0.03^d^	7.1 (7.1 to 7.2)	−0.00	−0.15	−0.01	0 (−0.02 to 0.02)	0.01	0.01	−0.01^d^	14.3 (14.3 to 15.0)
Sugar sweetened beverage consumption	−0.12	1.81	−1.14	65.9 (65.5 to 66.3)	0.13	0.63	−0.17^c^	31.0 (30.9 to 31.0)	0.02	0.58	−0.05	20.0 (19.9 to 20.1)	0.02	0.22	−0.03^c^	28.6 (28.56 to 28.59)
Last dental visit	−0.02	0.03	−0.01	0.6 (0.5 to 0.7)	0.02	0.12	−0.05^d^	4.8 (4.7 to 4.8)	0.01	−0.09	0.00	10.0 (9.98 to 10.02)	0.02	0.02	−0.03^c^	28.6 (28.55 to 28.58)
Water fluoridated area	0.04	0.05	−0.06	3.5 (3.1 to 3.8)	0.04	−0.05	−0.03	9.5 (9.5 to 9.6)	0.04	0.07	−0.09^e^	40.0 (39.9 to 40.1)	0	0.03	−0.01^e^	0 (−0.01 to 0.01)
Total	0.08	−0.01	−0.73	100	0.41	0.12	−0.48	100	0.10	0.26	−0.17	100	0.08	0.05	−0.11	100

Abbreviations: C, coefficient; E, endowment; I, interaction.

^a^ Following the work of Neumark,^1^ the coefficients were obtained from the pooled data regression.

^b^ Missing values for equivalized household income: all children, 13.5%; Indigenous children, 29.7%; and Non-Indigenous children, 11.7%.

^c^ *P* < .001.

^d^ *P* < .01.

^e^ *P* < .05.

## Discussion

The study aims were to characterize area- and individual-level oral health inequalities among Indigenous and non-Indigenous Australian children and to assess the contributions associated with individual-level modifiable risk factors on area-based inequalities in untreated dental caries. Although modifiable risk factors that were most strongly associated with the gap in untreated dental decay explained by school-level socioeconomic status for both Indigenous and non-Indigenous children were largely the same (consumption of SSBs, household income, residing in an area with water fluoridation), different risk factors were also observed. Examples of these factors were tooth-brushing frequency for Indigenous children and last dental visit for non-Indigenous children. The hypothesis that the percentage contribution associated with each risk factor would differ for Indigenous and non-Indigenous children proved true; there were substantial differences in the contributions associated with each of these risk factors. The findings have important policy translation implications, as they indicate that while targets to reduce consumption of SSBs may reduce oral health inequalities for all Australian children, an additional focus on oral hygiene is required for Indigenous children. This is important for both targeting of effective oral health promotion initiatives and for policy implications in the allocation of scarce resources in the public dental health setting.

It is important to comment on the overwhelming individual- and area-based oral health inequalities experienced by Indigenous children relative to their non-Indigenous peers. Irrespective of dentition, levels of untreated dental caries was 3 times higher among Indigenous children, which was not mitigated by area-based social advantage (Indigenous children attending the most advantaged schools experienced the same frequency of dental caries as non-Indigenous children in the least advantaged schools). This is contrary to evidence suggesting that positive oral health outcomes are associated with high socioeconomic status areas^20^ and suggests that, in the Australian context, there is something inherently unique in the social composition of Indigenous Australians that makes them more vulnerable to chronic disease health states (oral health is just 1 example) over and above indicators of social advantage.^21^

Child oral health inequalities provide insight into social inequality in a given society,^9^ and Australia is not alone in its efforts to ameliorate Indigenous-related health inequities.^22,23,24^ Scholars and community leaders have insisted that Indigenous health needs to be considered as separate from the health of other racial and ethnic minority groups within a given country, owing to the sustained colonization, discrimination, and marginalization, along with policies that focus on assimilation and, in some cases, cultural annihilation experienced by these groups.^25^ While our findings reflect health system differences (such as differences in preferential access for non-Indigenous groups, often due to many Indigenous Australian individuals residing in geographically remote locations with limited access to dental services), they also reflect a maldistribution of the social determinants of oral health; that is, Indigenous and non-Indigenous inequalities in education, jobs, material security, and experiences of discrimination. Attitudinal and cultural barriers also play a role,^26^ for example, a lack of cultural awareness among non-Indigenous health professionals, Indigenous suspicion (and sometimes rejection) of Western health systems,^27^ and the absence (in Australia) of treaty-based or formal recognition of Indigenous rights, including native title. Until only recently, substantial restrictions have been imposed on Indigenous Australian’s civil rights, residence, mobility, and employment.^28^

Untreated dental decay is an indicator of inability to access timely, appropriate, acceptable, and affordable dental care. The profound inequalities demonstrated indicate that Indigenous Australian children are not only disadvantaged with respect to dental disease, but also in their ability to access appropriate care for that disease. Untreated decayed teeth reflect not only unequal access to care, but also unequal social conditions (upstream of the dental profession), which lead to dental decay in the first instance.

### Limitations

It is important to discuss the study limitations. The design was cross-sectional, meaning no causal inferences can be implied. The water fluoridation estimates were based on carer-provided residential histories, with only the current place of residence used to characterize exposure to water fluoridation. This could have led to some misclassification. The study also has some substantial strengths, including being a large, representative sample of Australian children (for both Indigenous and non-Indigenous populations) that implemented internationally accepted measures for both parental self-reported information and clinical assessments, a nationally used school-based social deprivation index, sophisticated weighting approaches, and complex multilevel and decomposition techniques.

## Conclusions

Our study provides new evidence on the magnitude of oral health inequalities experienced by Indigenous and non-Indigenous Australian children and the contributions of individual-level modifiable risk factors on area-based inequalities in untreated dental caries for these groups. Efforts by the dental profession—as well as policy makers and health professionals more generally—are required at both national and international levels to reduce barriers to access to and the availability of preventive and rehabilitative oral health services for Indigenous groups. As reported in the Geneva Declaration on the Health and Survival of Indigenous Peoples,^29^ reducing oral health inequalities among and between Indigenous groups needs to be a public health priority at a global level.
